# Health-related quality of life in South African patients with pulmonary tuberculosis

**DOI:** 10.1371/journal.pone.0174605

**Published:** 2017-04-20

**Authors:** Tanja Kastien-Hilka, Bernd Rosenkranz, Edina Sinanovic, Bryan Bennett, Matthias Schwenkglenks

**Affiliations:** 1 Swiss Tropical and Public Health Institute (Swiss TPH), Basel, Switzerland; 2 University of Basel, Basel, Switzerland; 3 Health Economics Unit, Faculty of Health Sciences, University of Cape Town, Cape Town, South Africa; 4 Division of Clinical Pharmacology, Faculty of Medicine and Health Sciences, Stellenbosch University, Cape Town, South Africa; 5 Fundisa African Academy of Medicines Development, Cape Town, South Africa; 6 Patient Centered Outcomes, Adelphi Values, Bollington, United Kingdom; 7 Institute of Pharmaceutical Medicine, University of Basel, Basel, Switzerland; 8 Epidemiology, Biostatistics and Prevention Institute, University of Zürich, Zürich, Switzerland; Postgraduate Institute of Medical Education and Research, INDIA

## Abstract

**Background:**

The evaluation of patient-reported health-related quality of life (HRQOL) in pulmonary tuberculosis (TB) contributes to a comprehensive understanding of the burden associated with this disease. The aim of this study was to assess the overall impact of TB on the health status and on single health domains identified in the WHO definition of health, including physical, mental and social health aspects.

**Methods:**

Four instruments for HRQOL evaluation were applied in a longitudinal multicentre study during six-month standard TB treatment in South Africa. These included the generic SF-12 and EQ-5D-5L, the disease-specific St. George´s Respiratory Questionnaire (SGRQ) and the condition-specific Hospital Anxiety and Depression Scale (HADS). Statistical analysis included significance testing, univariable and multivariable analysis, and repeated measures ANOVA. Change over time in the physical component score (PCS) of SF-12 was defined as primary endpoint. A target sample size of 96 patients was estimated.

**Results:**

HRQOL of the study participants was impaired in all physical, mental and psycho-social health domains at treatment start. HRQOL improved significantly and in a clinically meaningful manner during the course of standard TB treatment, over the period of the study. The greatest improvement (95%) was observed in mental health. Younger patients with higher education and who were employed had a better HRQOL.

**Discussion:**

This study demonstrates the need for an integrative understanding of TB with HRQOL as core element to inform gaps in current TB management. Improvements in the management of TB following an integrative patient-centred approach will contribute towards meeting the United Nations Sustainable Development Goal 3 (SDG3) target and will support the End TB strategy of the WHO.

## Introduction

At the beginning of 2016, the United Nations (UN) introduced the Sustainable Development Goals (SDGs) to replace the Millennium Development Goals (MDGs) established in 2000 [1]. The third goal of the SDGs (SGD3) aims to ensure healthy lives and promote well-being at all ages [1]. One target of SGD3 focuses on universal health coverage, including access to safe, effective, high quality and affordable essential medicines [2]. Access to medicines requires sponsors to demonstrate that the medicine is safe, effective and affordable. The evidence of the real benefit of a treatment is usually evaluated based on clinical trials and real world data evidence. Only after this evidence has been provided, can medicines be made accessible through healthcare organizations. The World Health Organization (WHO) claims that patient involvement in their healthcare is a social, economic and technical necessity [3]. The evaluation of a patient-reported perspective of a disease and treatment contributes to a comprehensive understanding of the benefit and risk associated with that disease. This is important as some concepts cannot be measured objectively. One specific patient-reported outcome (PRO) is health-related quality of life (HRQOL). HRQOL is a PRO which refers to the multi-dimensional nature of health, and usually includes physical, mental and social health domains [4].

A further target of the UN SDG 3 includes an end to the epidemic of tuberculosis by 2030 [5]. Tuberculosis (TB) places a significant burden on the health system of South Africa, which has the highest prevalence and incidence rates of all 22 countries with a high burden of TB worldwide [6].

The impact of TB on HRQOL has been reported in a systematic literature review (Kastien-Hilka et al 2016). This systematic review found that TB had a negative impact on patients' HRQOL and overall wellbeing. The review further identified several factors associated with HRQOL in TB which included socio-demographic (age, gender) and socio-economic (income, education, housing, social security) characteristics, disease-related (symptoms) and therapy-related (side effects, adverse events) factors, and psycho-social aspects (isolation and stigmatization, psycho-social burden) [7–28]. Significant physical impairment caused by somatic symptoms and consequences of TB has also been reported [14, 23, 27, 29].

Although TB treatment results in a significant improvement in HRQOL, especially in physical and psychological dimensions [9, 10, 13–15, 18, 19, 23, 24, 26] the treatment regimen can be difficult for patients due to adverse drug reactions, the quantity of pills and treatment duration. This may have a significant impact on levels of adherence in this patient population. There is also some published evidence to suggest that amongst TB patients, psycho-social burden may have a greater impact than clinical symptoms. Specifically, anxiety and depression are the most frequently reported mental disorders reported in TB patients [30, 31]. It is believed that psychological distress may be caused by social stigmatization because of public misconception that a TB patient suffers from HIV co-infection [32, 33]. This association with HIV/AIDS may lead to a perception of social isolation during TB treatment and further impacted by the poor financial situation of many patients [27, 30, 34].

The impact of TB on HRQOL in South African populations was assessed in two studies only, [18, 35, 36]. As most other published studies addressing HRQOL in TB patients, both studies followed a cross-sectional study design. Data about longitudinal changes in HRQOL which were assessed in South African TB patients were not available when our study was planned and initiated. Since information on HRQOL contributes to efficient decision making, product approval, pricing and reimbursement as well as health policy making, HRQOL data will support the identification of sustainable health innovations in TB. A recognized reliable and validated TB-specific HRQOL measure has not been available, however all relevant HRQOL aspects of TB need to be captured to assess the achievement of the WHO´s End TB Strategy pillars.

The aim of this research was to evaluate patient-reported HRQOL in pulmonary TB in South Africa. The study sought to understand the overall impact of TB on the health status and on single health domains identified in the WHO definition of health, including physical, mental and social health aspects. The study addressed the impairment in HRQOL associated with TB, prior to treatment and longitudinal changes in HRQOL over the course of a six-month standard TB treatment.

## Methods

A detailed description of the study design and methodology has been published previously (Kastien-Hilka et al 2016). Briefly, the study followed an observational longitudinal design including prospective, repeated measures of HRQOL per study participant.

### Patient population and participant recruitment

Study participants were recruited between November 2014 and May 2015 at six selected primary health care clinics with the highest TB caseloads per month in Cape Town. Four of these facilities are run by the local government, and two by the provincial government, and are located in the Khayelitsha sub-district of the Cape Town Metro district. The sub-district has the highest TB burden in Cape Town, based on latest available caseloads from 2012 provided through the Western Cape Province. The study population comprised of patients diagnosed with active pulmonary TB. TB patients who were 18 years or older and were not diagnosed with TB before were eligible. Patients were excluded if they were diagnosed with multidrug-resistant TB (MDR-TB) and extensively drug-resistant TB (XDR-TB), and/or had HIV co-infection. Patients underwent HIV testing when diagnosed with TB before starting treatment. The TB nurses of each study site provided to the study team access to the HIV test results during the recruitment process to ensure a negative HIV status. HIV testing was not repeated during the treatment and any change in the HIV status was therefore unknown. The eligibility status of each patient was subject to verification by the nurse dedicated to TB patients of each health facility. Based on a patient information document, eligible patients were informed about the nature, purpose, potential risks and benefits of the study. Patients had the opportunity to decline their participation or to withdraw from the study at any time point. Patients who agreed to participate in the study signed a written informed consent.

Recruitment of new TB cases without HIV co-infection was difficult as a high proportion of TB patients are also HIV co-infected in South Africa. In order to meet the sample size in the pre-determined time frame, one additional clinic in a neighboring district was included in the study.

### Study procedures

Eligible participants received a six month standard TB treatment with rifampicin, isoniazid, ethambutol and pyrazinamide. Standard treatment comprised the intensive treatment phase over the first two months where all four antibiotic drugs were applied, and the continuous treatment phase with isoniazid and rifampicin only over at least additional 4 month. This standard treatment was not influenced by the study design. HRQOL was evaluated during the treatment course. The data collection regimen to monitor changes in HRQOL included five different time points over the six-month treatment period: beginning of treatment (baseline) and at follow-up visits after 4, 8, 16 weeks and after six-month treatment. The last data collection point at six months was selected as this time point represents the minimum treatment time for standard TB treatment. Data collection at treatment start and after six-months of treatment allowed the identification of HRQOL impairment caused by TB and any changes in HRQOL due to treatment. Since standard treatment comprised two treatment phases, data were collected at the switch between both phases (after 8 weeks) as well as in the middle of each treatment phase (after 4 weeks and 16 weeks). Data collection started when TB patients visited the study sites for treatment initiation; four HRQOL measures and one socio-demographic questionnaire were applied at baseline (treatment start). These four HRQOL measures were re-applied at all follow up visits. All HRQOL measures were applied in English language and have been validated for English for South Africa. No other languages or translations of these measures were applied. Data were collected based on completion of paper questionnaires during face-to-face interviews conducted by trained field workers. Field workers were trained and quality of data was ensured based on a Standard Operating Procedure, and details were published previously. Questionnaires were scanned by the field worker for any missing responses during the interviews.

#### Study materials

The rationale for the selection of HRQOL measures has been described previously (Kastien-Hilka et al 2016). Two generic (European Quality of Life 5 Dimensions 5 levels; EQ-5D-5L and Short-Form 12 items; SF-12), one disease-specific (St. George´s Respiratory Questionnaire; SGRQ) and one condition-specific (Hospital Anxiety and Depression Scale; HADS) HRQOL measures were used. All HRQOL measures have been validated in TB populations: SF-12 [18], EQ-5D-5L [37], SGRQ [38], and HADS [39].

#### EQ-5D-5L

EQ-5D-5L [40] is widely used as a utility index for estimating QALYs in cost-effectiveness studies [40, 41]. It comprises five items/domains (5D) (Mobility, Self Care, Usual Activities, Pain/Discomfort, and Anxiety/Depression) with each domain having five levels (5L): no problems, slight problems, moderate problems, severe problems, and extreme problems; The EQ-5D-5L includes further one vertical visual analogue scale (VAS 20 cm). Index-based values (utilities) are calculated from EQ-5D-5L by applying country-specific valuation algorithms. No South African specific valuation algorithms were available. Therefore, algorithms developed for the UK and for Zimbabwe, the only African country with an available algorithm, were used in this study. The EQ-5D-5L utilities range from 0 to 1, with higher scores indicating better health. A minimally important difference (MID) is only known for the 3 level version of EQ-5D, with a MID of 0.074 (range -0.011–0.140) and a MID of 7 for VAS scores [42]. EQ-5D-5L has an improved sensitivity compared to the 3 level version and its MID is also assumed for the 5 level version.

#### SF-12

Short-Form 12 (SF-12) is an abbreviated version of SF-36 containing 12 items representing eight domains (physical functioning, role physical, bodily pain, general health, vitality, social functioning, role emotional, and mental health) [41, 43]. Domains are aggregated into composite summary scores, Physical Component Score (PCS-12) and Mental Component Sore (MCS-12). Scoring ranges from 0 to 100 with greater scores representing better HRQOL. A score between 47 and 53 reflects normal scores for both PCS and MCS based on US population norms [44, 45]. No population norms for South Africa or other African countries are available. A MID of at least 3 points has been suggested for the SF-36 [44] and can also be used for SF-12v2 [46].

#### St. George´s Respiratory Questionnaire

St. George´s Respiratory Questionnaire (SGRQ) is a disease-specific instrument designed to assess patients with respiratory tract and immune system diseases, especially asthma, pulmonary diseases, and chronic obstructive disease [41, 47]. SGRQ comprises 50 items in three domains (Symptoms, Activity, and Impacts on daily life). Scores are scaled from 0 to 100, with higher scores indicating worse HRQOL. A MID for SGRQ is defined as an improvement of 4 points in the domain scores and the total score [48].

#### Hospital Anxiety and Depression Scale

Hospital Anxiety and Depression Scale (HADS) is a condition-specific instrument applied in psychology and psychiatry to detect states of anxiety and depression [49]. HADS comprises 14 items in two domains, Anxiety domain and Depression domain. The scores of each subscale range from 0–21 (8–10 mild, 11–14 moderate, 15–21 severe). A MID for HADS is not available from the developer. A MID of 1.5 points has been estimated for chronic obstructive pulmonary disease (COPD), corresponding to a change from baseline of 20% and informed by both anchor- and distribution-based methods [50].

### Sample size

The primary endpoint was defined as change in mean score of PCS-12 between baseline and six-month treatment. As SF-12 and SF-36 are comparable measures, sample size determination was based on the change in mean score of PCS-36 as reported by Walter [51]. We assumed a 4.0 point change in PCS-12 mean score (higher than the MID of 3 points) between baseline and visit 4 (six-months treatment) [45, 52], and a corresponding standard deviation (SD) of 7.0 for the mean score after six-months treatment [45], resulting in a standardized effect size of 0.57. Using the standardized effect size with a two-sided 5% level of significance and a 95% power yielded in an estimated sample size of n = 80 participants. We estimated an attrition rate of 20% resulting in a final sample size of n = 96 participants.

### Ethics

The study adhered to the International Conference on Harmonisation of Technical Requirements for Registration of Pharmaceuticals for Human Use (ICH) guidelines, the Declaration of Helsinki and South African Good Clinical Practice (GCP). The study was approved by the institutional review commission of the Swiss Tropical and Public Health Institute in Basel, Switzerland. Ethical approval was obtained from the ethical commission of North-West and Central Switzerland (EKNZ) and the ethics committee of the University of Cape Town. City Health of the City of Cape Town and the Western Cape Government of South Africa gave institutional approval of the study.

### Statistical analysis

Missing interviews were recorded as the absence of the study participant at the appointed interview time frame. Missing data were recorded when the interviewed participant did not complete HRQOL measures, or only insufficiently, hindering calculation of a domain or total score. Significance testing (paired-samples t-test), multivariable analysis and repeated measures ANOVA were based on observations that contained all data points required for a specific analysis.

All HRQOL responses were transferred to an excel-based database and all measures were scored according to each measure´s instructions.

EQ-5D-5L utility scores were calculation based on valuation algorithms for the UK and Zimbabwe, applying the EQ-5D calculator [40]. SF-12 component scores PCS-12 and MCS-12 as well as SGRQ domain and total score were calculated applying specific scoring software provided through the measure´s developer. HADS was scored based on an excel worksheet according to the instructions in the HADS manual [53].

Interpretation of EQ-5D-5L domain scores and HADS Anxiety and Depression domains were both based on categories: EQ-5D-5L on its five levels (no problems, slight problems, moderate problems, severe problems, and extreme problems) and both HADS domains on three levels (mild, moderate, severe). The SF-12 component scores and EQ-5D VAS were both interpreted based on a range from 0 (worst health) to 100 (best health), the EQ-5D-5L utility index on a range from 0 (worst health) to 1 (best health). The SGRQ domains and total score were interpreted based on a range between 0 (best health) to 100 (worst health) and compared to scores derived from a population with no history of respiratory disease [47].

Descriptive statistics were applied to socio-demographic data; further to all HRQOL data at baseline and at all follow-up visits to understand the HRQOL impairment of TB patients. Descriptive statistics were conducted including frequencies (N, N missing, %), central tendency (mean, median), and confidence interval (set at 95%). Distribution of data was examined by standard deviation (SD), minimum and maximum values, and frequency plots.

Overall changes in HRQOL between baseline and six-month treatment (visit 4) were calculated as frequencies (%). Longitudinal changes were determined by the change in mean scores between all follow-up visits and baseline. The change in mean scores was examined by paired-samples t-test with a statistical significance (2-tailed) set a priori at P < 0.05. Changes in mean scores in the intensive treatment phase (baseline to visit 2) were compared to changes in the continuous treatment phase (visit 2 to visit 4) based on paired-samples t-test with a statistical significance (2-tailed) set a priori at P < 0.05. The change in mean scores at each time point from baseline was also compared to the reported minimally important difference (MID) for each measure to understand if the longitudinal changes in HRQOL were clinically meaningful. Paired-samples t-test was applied to examine the difference between changes in mean scores and MID at a significance level of P< 0.05.

Differences in HRQOL mean scores among all HRQOL measures over time (baseline and follow-up visit 1–4) were examined by repeated measures ANOVA. Bonferroni correction was applied to repeated measures ANOVA for multiplicity of tests.

Responsiveness over time for each HRQOL was measured as an effect size partial eta squared providing information of the effect of time on changes in HRQOL aspects. The time effects were based on tests of within-subjects effects. When Mauchly´s test of sphericity was not met (significance < 0.05), both partial eta squared and observed power were derived from the Greenhouse-Geisser correction.

The impact of socio-demographic factors including age, gender, educational status and work status were elaborated. At baseline univariable analysis was applied to understand which factors might be associated with HRQOL, by using a cut off of P = 0.2. Resulting candidate factors were further assessed in multivariable models to understand the impact of socio-demographic factors over time (change from baseline to six month treatment (visit 4)). The univariable and multivariable analysis included a general linear model and an analysis of variance (ANOVA). The time effect of socio-demographic factors based on an effect size partial eta squared was further included by applying repeated measures ANOVA. Threshold values for the effect size were derived from Cohen [54]: 0.1 was interpreted as small, 0.3 as medium, 0.5 as large and 0.8 as very large.

Robustness of the findings was assessed by sensitivity analyses by excluding HRQOL data from visit 3. Further, the assessment of longitudinal changes of HRQOL over time including baseline and all follow-up data as described above was repeated by excluding data from visit 3, which was affected by a very low number of available observations. Results were checked for consistency.

## Results

In total, 131 eligible patients were recruited and agreed to participate in the study. Overall, 444 interviews were conducted over the duration of the study with questionnaires completed for data analysis.

From 131 participants who had baseline interviews conducted, 84 (64%) participated in the follow-up interview at visit 1, 85 (64%) participated at visit 2, 48 (36%) participated at visit 3 and 96 (70%) participated at visit 4. Only 20% of participants completed all HRQOL questionnaires at all time points during treatment as per protocol (Table 1).

**Table 1:** Overview of interviews taken per study site.

Data Collection Point	Total Number Interviews	Number of interviews						
		Study site 1	Study site 2	Study site 3	Study site 4	Study site 5	Study site 6	Study site 7
**Baseline (treatment start)**	**131**	**54**	**9**	**18**	**9**	**8**	**13**	**20**
**Visit 1 (4 weeks post treatment start**	**84**	**31**	**8**	**17**	**5**	**6**	**3**	**14**
**Visit 2 (8 weeks post treatment start**	**85**	**34**	**8**	**18**	**2**	**5**	**9**	**9**
**Visit 3 (16 weeks post treatment start**	**48**	**7**	**10**	**17**	**1**	**1**	**7**	**5**
**Visit 4 (24 weeks post treatment start**	**96**	**41**	**9**	**17**	**3**	**2**	**9**	**15**
**Total**	**444**							

During the course of treatment a total of 47, 46, 83, and 35 patients did not participate in follow-up interviews 1 to 4, respectively. In the majority of cases the reason for absence of participants was unknown. Of those participants who attended an appointment during the study period, none refused to participate in the interview. However, four patients were unable to participate at follow-up interviews as they had been transferred to a different hospital not included in this study and one patient received a custodial sentence. The lowest attendance rate to interviews was observed after 16 weeks of treatment (visit 3). The reasons why participants did not attend their clinic appointments, and hence the low response rate, was unknown. Although Direct Observed Treatment (DOT) is officially implemented to ensure patient compliance in South Africa, patients did not take medication under DOT; patients only visited the clinics once a month to collect their medication in advance. The interviews for HRQOL evaluation were taken, when a patient planned to visit the clinic for medication collection. When a patient did not visit the clinic at the scheduled medication collection date, the patient collected the medication at a different day. As communication to patients in this socio-demographic environment was very restricted due to the availability of mobile phones, a new interview appointment could only be re-scheduled in some cases.

In total, nine questionnaires were not completed by patients during the fieldwork. Whilst completing the sections on HRQOL measures, two patients did not answer the socio-demographic questionnaire, but all HRQOL measures; these two patients were still included in the data analysis. One of each measure was not answered during the data collection: SF-12 at baseline and at visit 1; EQ-5-D-5L at baseline and at visit 4; SGRQ at baseline, and HADS visit 1 and visit 2.

### Description of the study population

Table 2 describes the study population. Of 131 study participants, two participants did not provide information on socio-demographic data; 129 participants were included in the description of the study population. Study participants who provided socio-demographic data (n = 129) comprised 64% (n = 82) men and 36% (n = 47) women, age ranged from 18 years to 80 years, with the majority of participants being between 20 and 40 years and with a mean age of 36 years. The majority of participants were black (90%), living alone (i.e. not married nor co-habitation; 82%), and high school education attainment (68%). The majority of the study participants (73%) were unemployed, students or pensioners. Only 21% of the study participants were aware about existing co-morbidities impacting their health, most of them reporting to be diagnosed with other respiratory diseases or diabetes.

**Table 2:** Socio-demographic description of study population.

Socio-demographic Factors	Study population		
	N	%	N missing
**Gender**	129		2
Male	82	63.6	
Female	47	36.4	
**Age**	129		2
Mean age	35.8		
Median age	31		
Min	18		
Max	80		
**Age groups**	129		2
18–30	60	46.5	
31–50	46	35.7	
>51	23	17.8	
**Ethnicity**	129		2
Black	116	89.9	
Other	13	10.1	
**Marital status**	129		2
Single/never married/divorced/separated/widowed	103	81.7	
Married/co-habitation	23	18.3	
**Education**	129		2
Primary school	30	23.8	
High school	86	68.3	
College or university	10	7.9	
**Employment status**	128		3
Employed/self-employed/own business	34	26.6	
Unemployed/student/pensioner	94	73.4	
**Co-morbidities**	27		104
Other respiratory diseases	13	48.1	
Diabetes	13	48.1	
Cancers	None reported		
Cardio-vascular diseases	1	3.7	
Unknown co-morbidities	None reported		

The characteristics of the full study population were similar to the characteristics of those patients who provided information at the last visit, after six months of treatment.

### HRQOL impairment of TB patients at treatment start

Based on each measure´s score interpretation the HRQOL of the study participants was impaired in all physical, mental and psycho-social health domains at treatment start (baseline) (Table 3).

**Table 3:** Baseline impairment of HRQOL in TB patients at treatment start.

HRQOL measure	HRQOL domaim	N (%)	Mean score (SD)	95% Confidence Interval	Median	Range		N missing (%)
						Min	Max	
**SF12**	**PCS-12**	129	35.930 (8.321)	34.477–37.376	33.990	16.100	57.090	2
	**MCS-12**	129	36.011 (11.487)	34.013–38.015	35.840	11.690	68.900	2
**EQ-5D**	**Mobility**	129	2.442 (1.117)	2.247–2.637	3.000	1.000	5.000	2
	**Self Care**	129	2.434 (1.131)	2.237–2.631	3.000	1.000	5.000	2
	**Usual Activites**	129	2.558 (1.224)	2.345–2.771	3.000	1.000	5.000	2
	**Pain&Discomfort**	129	2.496 (1.098)	2.305–2.687	3.000	1.000	5.000	2
	**Anxiety&Depression**	129	2.566 (1.224)	2.353–2.779	2.000	1.000	5.000	2
	**Utility Index (UK)**	129	0.505 (0.328)	0.448–0.562	0.546	-0.594	1.000	2
	**Utility Index (Zim)**	129	0.620 (0.203)	0.584–0.655	0.621	-0.145	0.900	2
	**VAS score**	129	54.512 (18.066)	51.364–57.659	50.000	10.000	100.000	2
**SGRQ**	**Symptoms**	126	41.333 (33.803)	35.349–47.317	45.419	0.000	100.000	5
	**Activities**	126	70.087 (30.868)	64.622–75.522	79.671	0.000	100.00	5
	**Impact**	126	61.542 (25.638)	57.003–66.081	66.798	0.000	100.00	5
	**Total score**	126	61.134 (23.045)	57.055–65.214	66.366	0.000	92.643	5
**HADS**	**Anxiety**	131	11.504 (5.772)	10.506–12.502	12.000	0.000	21.000	0
	**Depression**	131	12.092 (6.237)	11.014–13.170	13.000	0.000	21.000	0

SF-12: 0 to 100 (= best HRQOL); EQ-5D domains: 1 = no problems, 2 = slight problems, 3 = moderate problems, 4 = severe problems, 5 = extreme problems. EQ-5D total index 0 to 1 (best HRQOL); EQ-5D VAS: 0 = worst health to 100 (= best health); SGRQ 0 to 100 (= worst health); HADS: normal (0–7) mild (8–10) moderate (11–14) severe (15–21)

### Development of HRQOL during TB treatment

**Fig 1:**
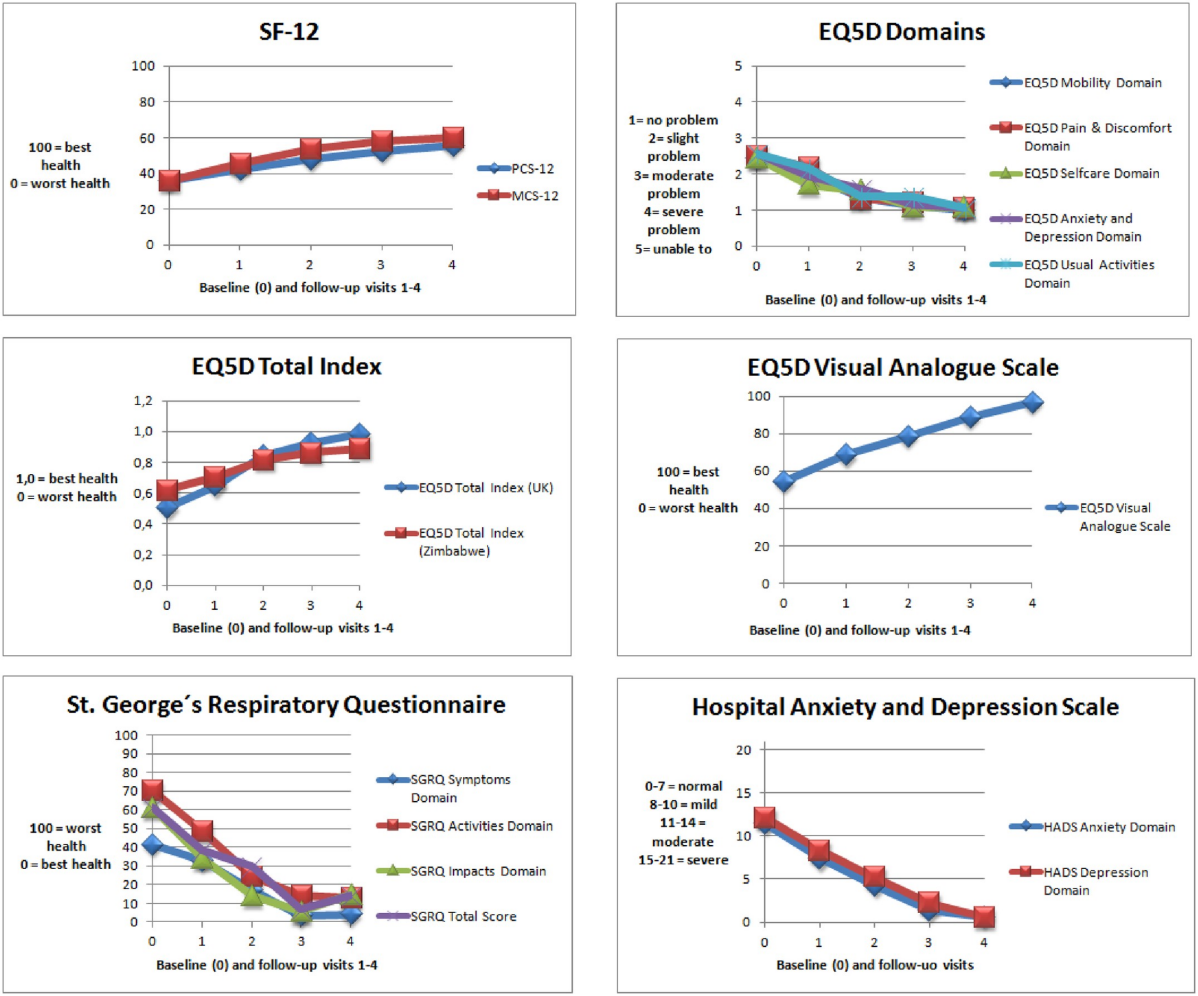
The development of HRQOL over six-month treatment.

**Fig 2:**
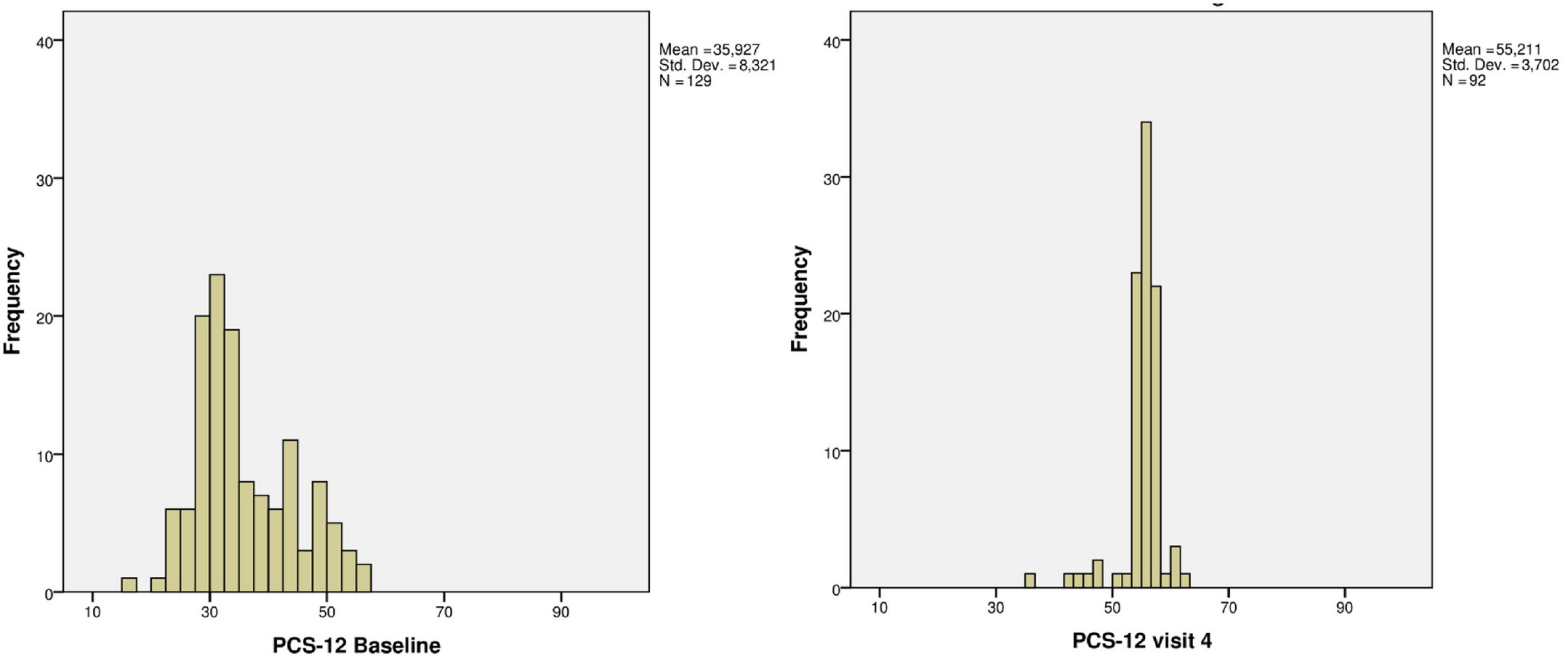
Histograms of PCS-12 at baseline and after six-month treatment.

**Fig 3:**
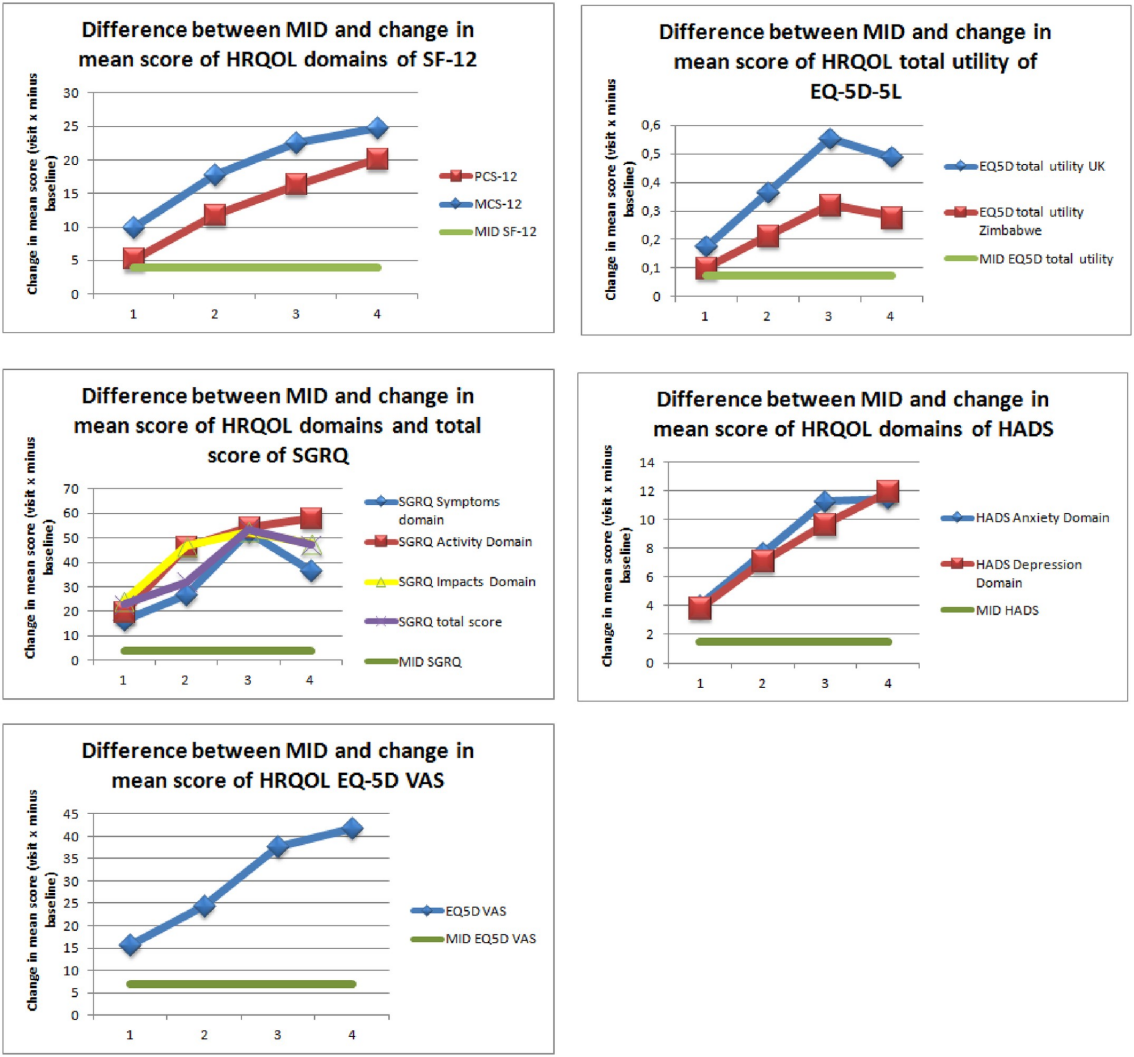
Change in HRQOL over six-month treatment.

All scores of HRQOL domains improved during the course of standard TB treatment, and over the period of the study (Fig 1). These changes were statistically significant. The largest improvement was observed in domains relating to mental health (increase in mean score between baseline and six month treatment of 59% - 95%), with the highest change observed in the HADS Anxiety and Depression domains, followed by improvements in the physical health domains and the social health domains. The primary endpoint defined as change in mean score of PCS-12 between baseline and six months from treatment initiation (visit 4) showed a change of 20 points (from 35.93 to 55.21) which was statistically significant (P <0.005) (Fig 2). The change in mean score was also greater than the published MID threshold to be considered clinically meaningful (Fig 3)

The change in HRQOL over time was statistically significant based on average mean scores. The change was greater than published MID thresholds for each HRQOL measure and thereby clinically meaningful. The MCS-12 mean score showed a greater relative improvement by +67% over six month treatment than the PCS-12 (+54%). Both component scores changed to a value of 55 points for PCS-12 and 60 points for MCS-12 and these values were above U.S. population norms (47 points for PCS-12 and 53 points for MCS-12) [44, 45]. All EQ-5D-5L average domain scores changed from an average state of “moderate health problems” to a state reporting “no problems” (+57–59%). The total index of EQ-5D reflected the same change as was observed on domain level. The total index calculated using the UK valuation algorithm changed by +94%. However, the total index value based on the Zimbabwe valuation algorithm changed by only +44%. The health status of the TB patients measured with the EQ-5D VAS changed by +78%.

The three SGRQ domains showed the greatest change in the physical symptoms domain with +90% improvement. Both domains of HADS (anxiety and depression) changed by +95% from a state of “moderate problems” to a state of reporting “no problems”.

The changes in mean scores during the intensive treatment phase were significant and (baseline to visit 2) higher than during the continuous treatment phase (visit 2 to visit 4). The changes in mean scores of the SGRQ Activities and Impact domains as well as in the SGRQ total score were non-significant during the continuous phase (S1 Table).

Changes in mean scores increased constantly until six month treatment for SF-12, EQ-5D VAS and HADS. The change in EQ-5D and SGRQ mean scores also increased significantly but with a peak after 16 weeks of treatment (visit 3) and changes in mean scores decreased until six month of treatment. Repeated measures ANOVA confirmed that the overall improvement in all HRQOL measures was associated with a statistically significant time effect ranging between 58% and 85%, with the highest effect observed on the health status (EQ-5D VAS) (S2 Table). Sensitivity analysis applied to repeated measure by leaving out data from visit 3 confirmed the robustness of the findings (S2 Table).

### The Influence of socio-demographic factors on baseline HRQOL and longitudinal changes in HRQOL

Significant results from the univariable analysis including the socio-demographic factors gender, age, educational level and work status were further assessed in a multivariable model.

The univariable analysis showed that HRQOL at baseline was more impacted in older patients with low educational background and without work. Age and education were significant factors in mental health domains, with younger patients with higher education reporting better HRQOL; work status played no major role in the mental health. With exception of MCS-12, gender played no role in HRQOL. Only in MCS-12 women reported a lower HRQOL than men. The multivariable model confirmed that younger patients (SGRQ Impacts domain, SGRQ total score, HADS Anxiety and Depression domains) with higher education (EQ-5D total index, HADS Anxiety and Depression domains) and with work (EQ-5D total index, SGRQ Symptoms domain) had a better HRQOL at baseline. These effects were only seen at baseline for all HRQOL measures (Table 4) and not after six months of treatment. PCS-12 mean scores at six months were not significantly different for gender, age, educational background and employment status.

**Table 4:** Associations between HRQOL and socio-demographic factors at baseline.

	EQ-5D utility index UK	EQ-5D utility index Zimbabwe	SGRQ Symptoms	SGRQ Impacts	SGRQ total	HADS Anxiety	HADS Depression
**Age**							
P value*	**-**	0.047	**-**	0.046	0.020	0.017	0.029
**18–30 years**	**-**	0.655	**-**	56.959	55.998	11.462	11.179
**31–50 years**	**-**	0.615	**-**	63.373	63.381	11.297	12.703
**>51 years**	**-**	0.501	**-**	75.168	74.813	15.588	15.824
**Education**							
P value*	0.015	0.015	**-**	**-**	**-**	0.010	0.044
**Primary school**	0.340	0.516	**-**	**-**	**-**	14.526	15.158
**High school**	0.528	0.629	**-**	**-**	**-**	11.862	12.154
**College or university**	0.745	0.766	**-**	**-**	**-**	7.286	9.000
**Work status**							
P value*	0.008	0.010	0.020	**-**	**-**	**-**	**-**
**Employed, self-employed, own business**	0.669	0.713	26.917	**-**	**-**	**-**	**-**
**Unemployed, student, pensioner**	0.444	0.578	46.399	**-**	**-**	**-**	**-**

* P values are for differences in baseline HRQOL between categories of socio-demographic factors.

Results from the repeated measures ANOVA showed no statistically significant effect of gender and age on the overall HRQOL improvement. However, education and work status showed significant effects. Patients with higher education and with work reported better HRQOL. Education (df = 1.000, F = 6.632, P = 0.012, eta = 0.074) and work status (df = 1.000, F = 7.789, P = 0.007, eta = 0.086) had a statistically significant but small effect on the HRQOL improvement with regard to PCS-12 (physical health). Similar effects were observed for the EQ5D utility indices (UK valuation algorithm: education df = 1.000, F = 7.071, P = 0.009, eta = 0.078; work status df = 1.000, F = 7.799, P = 0.006, eta = 0.085); Zimbabwe valuation algorithm: education df = 1.000, F = 6.821, P = 0.011, eta = 0.075; work status df = 1.000, F = 7.438, P = 0.008, eta = 0.081). Further, work status showed a statistically significant (df 1.000, F = 7.654, P = 0.007, eta = 0.081) but small effect on the overall improvement of the HRQOL domain SGRQ Symptoms (physical health) (S3 Table).

## Discussion

The increasing prevalence of non-communicable diseases (NCDs) and communicable diseases such as TB in South Africa leads to an increased pressure on healthcare resources. More importantly, from the patient perspective it also has an impact on the general well-being and HRQOL of patients.

This study was designed to evaluate changes in HRQOL in South African patients recently diagnosed with TB (without HIV co-infection) during the course of standard TB treatment over a six-month period. Two generic HRQOL measures (SF-12 and EQ-5D), a disease-specific measure (SGRQ) for respiratory diseases and a condition-specific measure for anxiety and depression, the HADS were used to assess HRQOL [55]. These four HRQOL measure captured relevant health aspects of TB in the physical, mental and psychosocial domains. The study population was recruited from the Cape Town district Khayelitsha, a socio-economically disadvantaged area. The majority of TB patients were in the productive years of their lives. However, more than 70% were not in employment. At the start of treatment, HRQOL domains and total scores on all measures indicated significant impairment in HRQOL. The results demonstrate that HRQOL improved significantly over treatment time, with the greatest improvement observed during the intensive phase of treatment and with greatest improvement in mental health domains.

Socio-demographic predictors for HRQOL at baseline included age, educational background and work status. Younger TB patients with a higher education, and who were in employment reported better HRQOL at baseline. A significant effect of educational background and work status has been reported before [56, 57]. It can be assumed that a better education increases the chances for work and financial security which might have a positive effect on HRQOL by improved self-care, improved social interactions, less psycho-social distress and less financial burden. Gender differences showed no significant effect on most HRQOL domains at baseline; only in the MCS-12 women reported a lower HRQOL than men. These effects of age, education and work status were not found after six months of treatment.

Gender and age were no predictors for positive changes in HRQOL over the treatment course. A recent study from Louw et al 2016 confirmed our findings of a non-effect of gender and age on HRQOL in TB patients after six month treatment in other Provinces in South Africa [58]. However, the same study reported that higher education was significantly associated with an improvement in mental health (MCS-12) after six month of treatment. Our study confirmed the effect of education on mental health aspects (anxiety and depression) only at baseline.

All changes over treatment time were statistically significant and were also clinically meaningful. The greatest improvements in physical health were measured with the respiratory-specific measure SGRQ. The primary endpoint defined as overall change in PCS-12 did not reflect the greatest improvements. It can be assumed that the Symptoms domain of SGRQ captures physical impairment due to TB in more detail and thereby being more sensitive to any changes in HRQOL than generic measures such as SF-12 and EQ-5D.

The study results revealed that the greatest improvement in all HRQOL domains was seen in the mental health of TB patients. The condition-specific HADS measure showed a similar impact on mental health at treatment start as reported from a study in Pakistan [59]. These results confirm previous findings suggesting that mental health is an important aspect in the treatment of TB. About 46–80% of TB patients in South Africa report common mental disorders [32, 35]. This psychological distress is associated with physiological TB symptoms, perceived health status, adverse events through anti-TB treatment and treatment outcome [30]. Furthermore, psychological distress may be caused by social stigmatization (HIV co-infection) followed by social isolation during TB treatment and impacted financial situation including fear of dying and disease transmission [27, 30–32, 34]. Special focus on reduction of stigmatization should be given in the management of TB to reduce the psychological distress [60]. This may suggest that patients perceive a positive outlook suffering less from the depression and anxiety associated with TB in South Africa. This again, is something that should be considered in the light of non-adherence. Inclusion of psychological support and treatment of mental disorders in the management of TB should be considered as well as interventions to reduce the stigmatization related to TB to improve the overall treatment outcome.

The physical and mental impairment seen in the South African patient population in this study is confirmed by studies in other countries applying the same HRQOL measures. The generic SF-12 measure indicated at treatment start a similar impairment in both the PCS-12 and MCS-12 with a score of 36, presenting defective physical functioning and a high risk for depression. A similar HRQOL impairment was reported in a study with TB affected immigrants in the UK [61] and from a study in Yemen [62]. Other studies which measured HRQOL by SF-36 or SF-12 in TB patients reported higher scores between 40 and 61 and thereby a better HRQOL at treatment start in Canada, Uganda, and Malaysia [24, 25, 37, 45, 52, 63]. Measuring the HRQOL in TB with the generic measure EQ-5D, our TB patients in South Africa reported a more impaired HRQOL and health status than in other countries (Malaysia, the UK, Canada) [14, 37, 61]. The same effect was found with the respiratory-specific measure SGRQ showing that our study population had a more impacted HRQOL than TB patients from Indonesia [64]. The SGRQ total score still showed some impairment with a score of 14 compared to a value of 5–7 reported by people from Spain with no respiratory history [47].

The phenomenon of an initial worsening in HRQOL after treatment start reported previously could not be confirmed by our observations [65]. Our study confirmed that the greatest improvement in HRQOL was seen during the intensive phase and this observation was reported before [9, 14, 15, 19, 26]. It is expected that monitoring of adherence during this treatment phase should be implemented to ensure that patients continue with their medication regime as many may perceive this improvement in their HRQOL as a sign of ‘cure’ and therefore no need to continue with their medication.

Comparing the results from the EQ-5D VAS and EQ-5D utility index based on value sets from UK with value sets from Zimbabwe clearly showed some cultural differences in HRQOL changes over time and its interpretation, and highlight the need for specific population norms for South Africa. The EQ-5D VAS reached a higher value of 97 than that of the general population in the UK of 82.5 and in Zimbabwe of 79.8 [66]. At baseline the utility index derived from the UK value set was by 0.115 points lower than the utility index derived from the Zimbabwean value set and this difference was greater than the MID for EQ-5D-5L. This is critically important in the context of the increasing request for cost-effectiveness analysis (CEA) from health systems in the allocation of resources. The EQ-5D is the preferred instrument to estimate population-based utilities for the calculation of quality-adjusted life years (QALYs) to provide a cost per QALY outcome in CEA. In 2013, the South African Department of Health published the Guidelines for Pharmacoeconomic Submissions which requests the use of QALYs for CEA [67]. The development towards pharmacoeconomic evaluations in South Africa for efficient decision making would require data acquisition to establish South African population norms for the EQ-5D.

### Limitations

This study had several limitations. HRQOL was only evaluated in patients with newly diagnosed pulmonary TB and patients with other forms of TB including multi-drug resistance (MDR-)TB were not included. If MDR-TB was diagnosed during treatment monitoring the patient had to change the treatment and was excluded from the study. If and at which time point drug susceptibility testing was performed at the different study sites was unknown. Furthermore, this study did not include testing of drug intolerance or drug sensitivity and the impact of drug intolerance and drug sensitivity on HRQOL was not known.

Our sample size was difficult to reach in one restricted district due to the low number of TB patients without HIV co-infection, as well as due to time and resource limitations in this study. In South Africa the high majority of TB patients are HIV co-infected; this study did exclude HIV co-infection to understand the isolated TB impact on HRQOL. Further, this study did not include a comparison or control group from the general population, for time and resource limitations. Our study did not apply a mixed method approach and was limited to a quantitative approach; patient perspectives about TB in a South African cultural background were not qualitatively assessed. Such data might have enriched the quantitative findings for HRQOL in this study. HRQOL was only assessed in a specific district of the Western Cape Province with a specific socio-demographic and –economic situation. Results from this study may not be generalizable to the rest of the South African population which is very diverse. Receiving treatment for their condition may have put an increased economic pressure on these participants as they may have been unable to take on work or consider their working options due to the travel distance between a health clinic they get the treatment from and workplace, but also due to the disease induced worsening of their physical condition [12, 27, 29].

Additionally, there is an increased risk of tuberculosis when diagnosed with diabetes [68], therefore we expected diabetes to be a major co-morbidity in our TB patients. We included questions about NCDs in our study, however it was apparent that only a small portion of TB patients were aware of any co-morbidity they may have, including diabetes. We assume that other health issues are unknown to a majority of TB patients. These unknown co-morbidities are assumed to have an additional impact on the HRQOL in TB patients at baseline which we could not distinguish.

## Conclusions

TB negatively impacts the HRQOL of patients, with specific impairment reported in physical, mental and psycho-social health aspects, however with treatment HRQOL improves significantly. Different aspects of health (HRQOL domains) are impacted differently and it would appear that the rate of improvement in each domain may also be different. HRQOL reveals different outcomes depending on the type of measure applied and depending on the cultural background and the study setting, making comparison of HRQOL outcomes difficult. Generic HRQOL PROs may not adequately capture all relevant aspects of TB, and thus a disease-specific HRQOL measure is required. This study also demonstrates the need for an integrative understanding of TB with HRQOL as core element to inform gaps in current TB management. Improvements in the management of TB following an integrative patient-centred approach will contribute towards meeting the UN SDG3 target and will support the End TB strategy of the WHO.

## Supporting information

S1 Table Changes in HRQOL in the intensive and continuous treatment phase. (DOCX)

S2 Table Overall treatment time effect on HRQOL (test of within-subjects effects). (DOCX)

S3 Table Effects of socio-demographic factors on overall HRQOL improvement. (DOCX)
