# Supplementary material for: Health-related quality of life in South African patients with pulmonary tuberculosis
Source: PLoS One. 2017 Apr 20;12(4):e0174605. doi: 10.1371/journal.pone.0174605 (PMC5398494; doi:10.1371/journal.pone.0174605)
Supplement: S1 Table — (DOCX) [file pone.0174605.s001.docx]

**Supplementary Material**

**Table S1. Changes in HRQOL in the intensive and continuous treatment phase.**

| **HRQOL** | **Treatment Phase** | **N (pairwise)** | **Change in mean** | **P value** |
| --- | --- | --- | --- | --- |
| PCS-12 | Intensive phase | 83 | 11.785 | < 0.05 |
|  |  |  |  |  |
|  | Continuous phase | 65 | 7.991 | < 0.05 |
|  |  |  |  |  |
| MCS-12 | Intensive phase | 83 | 17.708 | < 0.05 |
|  |  |  |  |  |
|  | Continuous phase | 67 | 6.644 | < 0.05 |
|  |  |  |  |  |
| EQ-5D index UK | Intensive phase | 84 | 0.365 | < 0.05 |
|  |  |  |  |  |
|  | Continuous phase | 67 | 0.152 | < 0.05 |
|  |  |  |  |  |
| EQ-5D total index Zimbabwe | Intensive phase | 84 | 0.213 | < 0.05 |
|  |  |  |  |  |
|  | Continuous phase | 67 | 0.083 | < 0.05 |
|  |  |  |  |  |
| EQ-5D VAS | Intensive phase | 85 | 24.482 | < 0.05 |
|  |  |  |  |  |
|  | Continuous phase | 69 | 18.681 | < 0.05 |
| SGRQ Symptoms | Intensive phase | 80 | 26.602 | < 0.05 |
|  |  |  |  |  |
|  | Continuous phase | 68 | 11.505 | 0.002 |
| SGRQ Activities | Intensive phase | 84 | 46.481 | < 0.05 |
|  |  |  |  |  |
|  | Continuous phase | 69 | 9.442 | 0.527 |
| SGRQ Impacts | Intensive phase | 84 | 46.953 | < 0.05 |
|  |  |  |  |  |
|  | Continuous phase | 70 | 4.246 | 0.767 |
| SGRQ total score | Intensive phase | 81 | 32.070 | < 0.05 |
|  |  |  |  |  |
|  | Continuous phase | 68 | 14.546 | 0.487 |
| HADS Anxiety | Intensive phase | 85 | 7.600 | < 0.05 |
|  |  |  |  |  |
|  | Continuous phase | 68 | 3.82 | < 0.05 |
| HADS Depression | Intensive phase | 85 | 7.071 | < 0.05 |
|  |  |  |  |  |
|  | Continuous phase | 68 | 4.779 | < 0.05 |
