# Supplementary material for: Health-related quality of life in South African patients with pulmonary tuberculosis
Source: PLoS One. 2017 Apr 20;12(4):e0174605. doi: 10.1371/journal.pone.0174605 (PMC5398494; doi:10.1371/journal.pone.0174605)
Supplement: S2 Table — (DOCX) [file pone.0174605.s002.docx]

**Supplementary Material**

**Table S2. Overall treatment time effect on HRQOL (test of within-subjects effects).**

|  | | | | | **Sensitivity Analysis baseline, 8 weeks and 24 weeks treatment** | | | | |
| --- | --- | --- | --- | --- | --- | --- | --- | --- | --- |
| **HRQOL** | **N** | **P < 0.05** | **Partial eta squared** | **Observed power** | **HRQOL** | **N** | **P < 0.05** | **Partial eta squared** | **Observed power** |
| PCS-12 | 26 | <0.05 | 0.686 | 1.000 | PCS-12 | 65 | < 0.05 | 0.748 | 1.000 |
| MCS-12 | 27 | <0.05 | 0.698 | 1.000 | MCS-12 | 67 | < 0.05 | 0.691 | 1.000 |
| EQ5D total index UK | 27 | <0.05 | 0.619 | 1.000 | EQ5D total index UK | 66 | < 0.05 | 0.621 | 1.000 |
| EQ5D total index Zimbabwe | 27 | <0.05 | 0.596 | 1.000 | EQ5D total index Zimbabwe | 66 | < 0.05 | 0.588 | 1.000 |
| EQ5D VAS | 28 | <0.05 | 0.845 | 1.000 | EQ5D VAS | 69 | < 0.05 | 0.808 | 1.000 |
| SGRQ Symptoms | 23 | <0.05 | 0.582 | 1.000 | SGRQ Symptoms | 65 | < 0.05 | 0.425 | 1.000 |
| SGRQ Activities | 28 | 0.286 | 0.043 | 0.192 | SGRQ Activities | 68 | 0.001 | 0.143 | 0.933 |
| SGRQ Impacts | 28 | 0.231 | 0.053 | 0.226 | SGRQ Impacts | 69 | 0.002 | 0.126 | 0.887 |
| SGRQ total score | 24 | 0.494 | 0.030 | 0.157 | SGRQ total score | 65 | 0.057 | 0.047 | 0.538 |
| HADS Anxiety | 27 | <0.05 | 0.653 | 1.000 | HADS Anxiety | 68 | <0.05 | 0.664 | 1.000 |
| HADS Depression | 27 | <0.05 | 0.604 | 1.000 | HADS Depression | 68 | <0.05 | 0.642 | 1.000 |

Greenhouse-Geisser Test was applied as sphericity was not assumed (P < 0.05).
