# Supplementary material for: Health-related quality of life in South African patients with pulmonary tuberculosis
Source: PLoS One. 2017 Apr 20;12(4):e0174605. doi: 10.1371/journal.pone.0174605 (PMC5398494; doi:10.1371/journal.pone.0174605)
Supplement: S3 Table — (DOCX) [file pone.0174605.s003.docx]

**Supplementary Material**

**Table S3. Effects of socio-demographic factors on overall HRQOL improvement.**

| **HRQOL** | **Source** | **Df** | **F** | **P value** | **Partial eta squared** |
| --- | --- | --- | --- | --- | --- |
| **PCS-12** | PCS-12 x gender | 1.000 | 1.047 | 0.309 | 0.012 |
|  | PCS-12 x Age Groups | 1.000 | 0.082 | 0.775 | 0.001 |
|  | PCS-12 x Education | 1.000 | 6.632 | 0.012* | 0.074 |
|  | PCS-12 x Work Status | 1.000 | 7.789 | 0.007* | 0.086 |
| **MCS-12** | MCS-12 x gender | 1.000 | 1.949 | 0.166 | 0.022 |
|  | MCS-12 x Age Groups | 1.000 | 0.000 | 0.999 | 0.000 |
|  | MCS-12 x Education | 1.000 | 0.880 | 0.351 | 0.010 |
|  | MCS-12 x Work Status | 1.000 | 1.341 | 0.250 | 0.015 |
| **EQ5D total index UK** | EQ5DindexUK x gender | 1.000 | 0.484 | 0.488 | 0.006 |
|  | EQ5DindexUK x Age Groups | 1.000 | 1.537 | 0.219 | 0.018 |
|  | EQ5DindexUK x Education | 1.000 | 7.071 | 0.009* | 0.078 |
|  | EQ5DindexUK x Work Status | 1.000 | 7.799 | 0.006* | 0.085 |
| **EQ5D total index Zimbabwe** | EQ5DindexZim x gender | 1.000 | 0.263 | 0.609 | 0.003 |
|  | EQ5DindexZim x Age Groups | 1.000 | 1.696 | 0.196 | 0.020 |
|  | EQ5DindexZim x Education | 1.000 | 6.821 | 0.011* | 0.075 |
|  | EQ5DindexZim x Work Status | 1.000 | 7.438 | 0.008* | 0.081 |
| **EQ5D VAS** | EQ5D VAS x gender | 1.000 | 0.190 | 0.664 | 0.002 |
|  | EQ5D VAS x Age Groups | 1.000 | 1.467 | 0.229 | 0.017 |
|  | EQ5D VAS x Education | 1.000 | 3.098 | 0.082 | 0.034 |
|  | EQ5D VAS x Work Status | 1.000 | 1.878 | 0.174 | 0.021 |
| **SGRQ Symptoms** | SGRQ Symptoms x gender | 1.000 | 0.217 | 0.642 | 0.003 |
|  | SGRQ Symptoms x Age Groups | 1.000 | 3.325 | 0.072 | 0.038 |
|  | SGRQ Symptoms x Education | 1.000 | 0.004 | 0.950 | 0.000 |
|  | SGRQ Symptoms x Work Status | 1.000 | 7.654 | 0.007* | 0.083 |
| **SGRQ Activities** | SGRQ Activities x gender | 1.000 | 0.435 | 0.511 | 0.005 |
|  | SGRQ Activities x Age Groups | 1.000 | 0.364 | 0.548 | 0.004 |
|  | SGRQ Activities x Education | 1.000 | 1.929 | 0.169 | 0.022 |
|  | SGRQ Activities x Work Status | 1.000 | 0.202 | 0.655 | 0.002 |
| **SGRQ Impacts** | SGRQ Impacts x gender | 1.000 | 0.702 | 0.404 | 0.008 |
|  | SGRQ Impacts x Age Groups | 1.000 | 0.462 | 0.498 | 0.005 |
|  | SGRQ Impacts x Education | 1.000 | 1.889 | 0.173 | 0.021 |
|  | SGRQ Impacts x Work Status | 1.000 | 0.638 | 0.427 | 0.007 |
| **SGRQ total score** | SGRQ total score x gender | 1.000 | 0.669 | 0.416 | 0.008 |
|  | SGRQ total score x Age Groups | 1.000 | 0.461 | 0.499 | 0.006 |
|  | SGRQ total score x Education | 1.000 | 2.271 | 0.136 | 0.027 |
|  | SGRQ total score x Work Status | 1.000 | 0.280 | 0.598 | 0.003 |
| **HADS Anxiety** | HADS Anxiety x gender | 1.000 | 0.470 | 0.495 | 0.006 |
|  | HADS Anxiety x Age Groups | 1.000 | 1.396 | 0.241 | 0.016 |
|  | HADS Anxiety x Education | 1.000 | 3.881 | 0.052 | 0.044 |
|  | HADS Anxiety x Work Status | 1.000 | 0.794 | 0.375 | 0.009 |
| **HADS Depression** | HADS Depression x gender | 1.000 | 0.302 | 0.584 | 0.004 |
|  | HADS Depression x Age Groups | 1.000 | 3.499 | 0.065 | 0.040 |
|  | HADS Depression x Education | 1.000 | 2.377 | 0.127 | 0.027 |
|  | HADS Depression x Work Status | 1.000 | 0.321 | 0.572 | 0.004 |

*Significant at P < 0.05.
